# Supplementary material for: First Report of Plasmid-Mediated Macrolide-Clindamycin-Tetracycline Resistance in a High Virulent Isolate of Cutibacterium acnes ST115
Source: Pathogens. 2023 Oct 27;12(11):1286. doi: 10.3390/pathogens12111286 (PMC10674219; doi:10.3390/pathogens12111286)
Supplement: Supplementary file 1 [file pathogens-12-01286-s001.zip › pathogens-2621913-supplementary.pdf]

pathogens

MDPI

Supplementary file

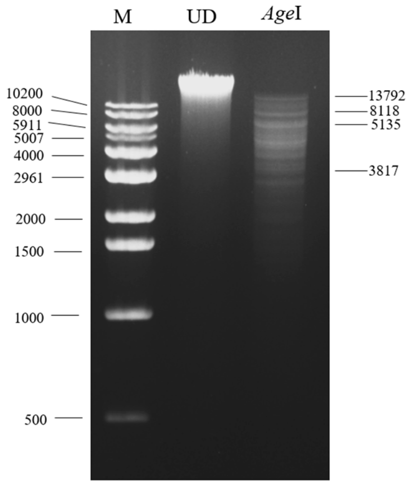

Supplementary Figure S1. Agarose gel electrophoresis of *C. acnes* KB112 plasmid DNA and restriction enzyme digestion. Lane 1: (M) 1-kb DNA ladder; Lane 2: (UD) Undigested plasmid DNA (30,947 bp); Lane 3: Digested by *Age*I restriction enzyme (expected size in bp: 13,792; 8,118; 5,135; 3,817, respectively).

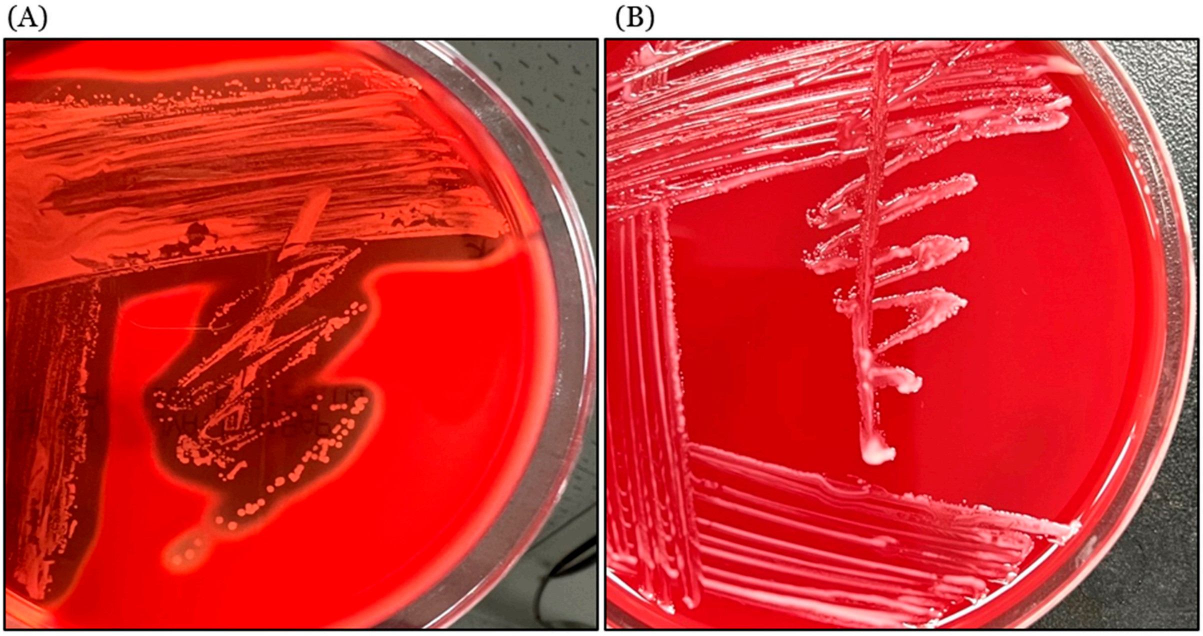

pathogens

MDPI

Supplementary Figure S2. Hemolysis of *C. acnes* KB112 and *C. acnes* ATCC 11828 on blood agar plate. Anaerobic incubation for 72 h at 37°C. (A) *C. acnes* KB112 shows hemolysis (clearing of blood agar) and (B) *C. acnes* ATCC 11828 shows no hemolysis.

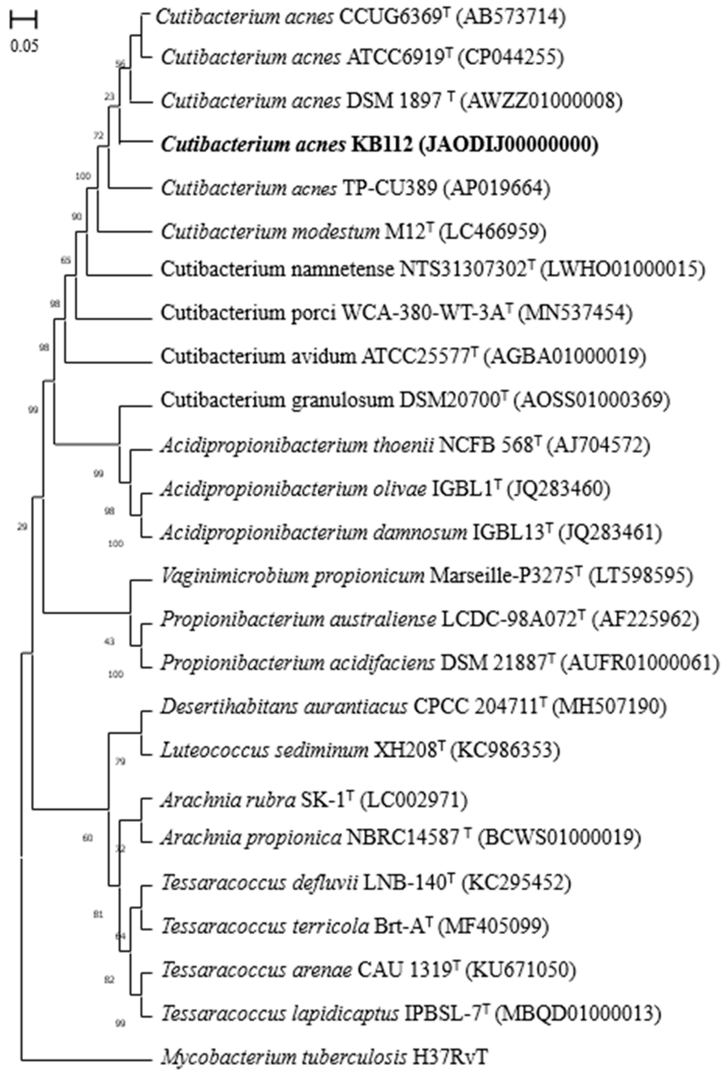

Supplementary Figure S3. The phylogenetic tree highlights the position of *C. acnes* KB112 in relation to several related bacterial strains. The tree was constructed using the Mega software and the neighbor joining approach. The scale bar shows the divergence times of various strains. The genome of *Mycobacterium tuberculosis* strain H37Rv (AL123456) was used as an outgroup.
